# Age Influences on Lifestyle and Stress Perception in the Working Population

**DOI:** 10.3390/nu15020399

**Published:** 2023-01-12

**Authors:** Daniela Lucini, Eleonora Pagani, Francesco Capria, Michele Galiano, Marcello Marchese, Stefano Cribellati, Gianfranco Parati

**Affiliations:** 1 BIOMETRA Department, University of Milan, 20129 Milan, Italy; 2 Exercise Medicine Unit, Istituto Auxologico Italiano, IRCCS, 20135 Milan, Italy; 3 Department of Psychology, Catholic University of the Sacred Hearth, 20123 Milan, Italy; 4 Assidim, 20122 Milan, Italy; 5 SEGE Srl, 20146 Milan, Italy; 6 Department of Medicine and Surgery, University of Milano-Bicocca, 20126 Milan, Italy; 7 Department of Cardiology, Istituto Auxologico Italiano, IRCCS, 20149 Milan, Italy

**Keywords:** age, lifestyle, exercise, prevention, workplace health promotion, gender, stress

## Abstract

Workplace health promotion programs and services offered by insurers may play a fundamental role to foster health/well-being and to prevent chronic diseases. To this end, they should be tailored to companies/employees’ requirements and characteristics. In particular, age needs to be taken into account, considering both that young age workers are generally healthy, and that young age is the best period in lifespan to address prevention and instilling healthy behaviors. We employed an anonymous, simple web-based questionnaire (filled out by 1305 employees) which furnishes data regarding lifestyle (nutrition, exercise, smoking, stress, sleep, etc.), some of which were used to build a unique descriptor (Lifestyle Index; 0–100 higher scores being healthier). We considered three subgroups accordingly to age: ≤30; between 30 and 50; >50 years. This study showed age influences lifestyle and stress perception in the working population: the youngest employees (both men and women) presented the worst lifestyle index, particularly in its stress component. This observation may potentially be useful to tailor workplace health promotion programs and to personalize insurance protocols and services offered to employees. The practical message of our study is that in healthy young people focusing only on medical parameters (frequently within normal ranges in this cohort), albeit important, may be not sufficient to foster proactive actions to prevent chronic non-communicable diseases in adult life. Vice versa, driving their attention on current behaviors might elicit their proactive role to improve lifestyle, getting immediate advantages such as well-being improvement and the possibility to best manage stress.

## 1. Introduction

Good health and well-being represent one of the World Health Organization’s goals for sustainable development [1]. The pursuit of a healthy lifestyle may actually be considered a real sustainable tool: to take action today (improving individual behavior) to preserve health which, otherwise, might disappear in the future [2]. Moreover, a healthy lifestyle contributes to the prevention of chronic non-communicable diseases (CNCD), granting benefits not only at the individual level but also at the global level, saving economic resources that might be necessary in order to manage those chronic diseases, and saving the planet’s resources [3,4]. The benefits of a healthy lifestyle overcome the prevention and management of CNCD being associated with an improvement of social relationships, stress management, a betterment of work and scholastic performances, absenteeism reduction, and increased productivity [3,5,6,7]. The importance to address lifestyle in the prevention and management of CNCD is also corroborated by the observation that social environments strongly influence individual behavioral choices henceforth affecting health [8]. Main international guidelines for the prevention/treatment of many CNCD [9,10,11] indicate the improvement in lifestyle as mandatory, clearly defining, for instance, the characteristics of a healthy lifestyle, the dose and modality of the required exercise or nutrition pattern to reduce overall cause mortality and to prevent CNCD. Additionally, guidelines to promote health and stress management in the workplace indicates a healthy lifestyle as a pivotal tool [12,13]. Nevertheless obesity, sedentariness and stress are increasing [14,15], particularly in youth. To move subjects/patients towards a behavioral change is very difficult and simple counseling is not always a winning strategy [16,17,18], even if the execution of diagnostic examinations is associated [19]. Successful interventions need to consider actions at community and individual levels [16,20,21] and different stakeholders may be involved. Educational institutions (such as school, and family), non-profit associations, companies, and workers organizations, may have a fundamental role acting at the community level, while medical institutions and professionals may best act at the individual level. In this context, insurers and organizations offering services to workers may play a particular role, furnishing from on one hand services at the community level aimed to educate people (educational campaigns, trainings, websites dedicated to health issues, etc.) and, from other hand, services aimed to improve health at individual level (medical assessments, diagnostic tests, and individual intervention programs). A major pitfall in this context may be represented by the cost, quality, and usefulness of proposed educational or intervention programs both at community and individual levels, which are often generic and delivered to the entire work population without considering even simple issues, for instance, age or gender (aside from programs geared to the prevention of gender-specific diseases such as breast or prostate cancer) [19]. The formula “one size fits all” is convenient from an organizational and cost point of view, but the return on investments is not always positive. On the other hand, a real personalized approach considering individual clinical characteristics and preferences in order to prescribe an intervention program to improve lifestyle [2,9,22] is obviously limited to a medical setting and rarely may be realized in the workplace.

The possibility to assess lifestyle in a friendly, cost-effective way at the workplace may facilitate the individualization of groups or subgroups of employees who deserve particular action in order to manage specific health issues or improve a distinct behavior. In a previous paper [23] we recently showed that perceptions of stress, fatigue, and somatic symptoms related to stress were higher in women that in men, using a simple anonymous web-based questionnaire, offering a simple, albeit potentially useful, metric to tailor workplace interventions [24,25,26].

Age is another important parameter to consider in order to plan interventions to foster a healthy lifestyle reducing the incidence of CNCD. Ideally, childhood and youthhood are the best periods in lifespan to address primordial prevention and instilling healthy behaviors [20].

Cardiovascular and cardiometabolic risk profiles are generally determined employing factors (such as blood lipids, blood pressure, glucose levels, body mass index (BMI), etc.) which are often within the normal range in the young population [9,15]. A more recent approach to the prevention of CNCD and to health promotion focuses more on lifestyle components than on traditional risk factors linked to clinical parameters, suggesting an important change of “point of view” [27]. Maintaining a healthy lifestyle or an early start of lifestyle changes is of paramount importance in order to optimize the prevention of CNCD in adults [20]. This implies a different methodology [21] which considers of paramount importance “how” to improve behavior and not only which factor (for instance blood pressure level or cholesterol level) needs to be changed.

Young adults represent a significant portion of the working-age population; they are very different from the previous generation, and also have different professional roles [28]. They represent an ideal population to consider in order to plan workplace intervention to foster health and reduce the future incidence of CNCD. On the contrary, their adherence to company policy and participation in planned screening (check-ups), frequently offered to all employees as part of a workplace policy to promote health, are lower than expected [29]. Vice-versa older adults seem to better comply (albeit in part) to planned screening, perhaps realizing the presence of initial signs or symptoms that may be associated with diseases.

The aim of the present study was to verify the potentiality of an anonymous, web-based questionnaire, to easily detect age differences in lifestyle, in order to tailor interventions to foster health at the worksite and, consequently, to reduce the risk of CNCD.

## 2. Materials and Methods

In total, 1333 employees of several Italian companies randomly filled out, on a voluntary basis, an anonymous questionnaire on lifestyle present from January 2021 to April 2021 in the web page of Assidim (a non-profit association that provides the associated companies, their employees, and families financial assistance and support in case of accident, disease, death, or invalidity) which considers, since its foundation in 1981, health promotion among workers and associated companies as its mission. We recently described [23] the methodology employed to create this questionnaire, which was validated by statistical analysis [24,25,26] and employed in some studies approved by ethical committees (for instance: Ethics Committee of University of Milan (No. KB 382/2017) (report dated 23 September 2019 and report dated 14 December 2021). Briefly, it is anonymous, it was designed [30] to obtain data on lifestyle (diet habits, exercise, smoking, sleep hours, alcohol, and perception of stress), on the working role, on the perception of quality of personal health, on sleep quality, on job performance, on the presence of chronic disease, and anthropometric data. Perception of quality of sleep and quality of health was assessed by providing nominal self-rated Likert scales from 0 (‘bad’) to 10 (‘very good’) for each measure. Perception of job performance was assessed providing nominal self-rated Likert scales from 0 (‘bad’) to 5 (‘very good’). The questionnaire, although anonymous, provided every single participant with a personalized immediate report based on the provided information.

Quality analysis of collected data [23] was conducted to cut out from the dataset non-realistic data and 98% of the questionnaires were finally included in the statistical analysis.

### 2.1. Lifestyle Assessment

As we publish in our previous papers (see references [23,24,25,26] for details), we assessed lifestyle considering the following items:

- Physical activity (weekly physical activity volume) [31,32] using the following equations to guess weekly physical activity volume: Moderate-intensity (MET·minutes/week) = (3.3 × minutes of brisk walking × days of brisk walking) + (4.0 × minutes of other moderate-intensity activity × days of other moderate-intensity activities); vigorous-intensity: (MET·minutes/week) = 8.0 × minutes of vigorous-intensity activity × days of vigorous-intensity activity; Total Weekly physical activity volume [MET·minutes/week] = sum of Moderate + Vigorous MET·minutes/week scores.

- Nutrition was guessed using the American Heart Association (AHA) Diet Score [33], adapted to Italian eating habits) [30].

- Perception of somatic symptoms (short 4SQ), fatigue and stress, were determined using a self-administered questionnaire [25,26] providing nominal self-rated Likert scales from 0 to 10 for each measure. Short 4SQ considers 4 somatic symptoms, and thus the total score ranged from 0 to 40.

Smoking behavior: all subjects who reported having never smoked or to have stopped smoking for more than one year were considered non-smokers.

In order to have a unique descriptor of lifestyle, as previously described [23], we took into consideration three domains: exercise (total activity dose), nutrition (combination of AHA Diet Score and WC), and stress (combination of scores of somatic symptoms, stress, and fatigue perception). The three domains were combined into a single Index of Healthy Lifestyle, which ranged from 0 to 100 (higher scores being healthier) using weights for measures of activity, diet, and stress according to our prior experience in a similar setting (see references [23,25] for more details).

All subjects voluntarily inserted anonymous data and they were aware of the possible use of group data for scientific purposes.

We subdivided the entire cohort into three subgroups accordingly to age: group 1: age ≤ 30 years; group 2: age between 30 and 50 years; group 3: age > 50 years.

### 2.2. Statistics

Summary data are presented as mean ± SD. Statistical significance of the differences between groups was evaluated with GLM (General Linear Model) considering age groups and gender as factors. Chi-square tests were used for categorical variables. Computations were performed with a commercial statistical package (IBM SPSS Statistics for Windows, version 27. IBM Corp., Armonk, NY, USA), using a PC (DELL). A *p* < 0.05 was taken as the threshold.

## 3. Results

We considered for analysis only the fully completed questionnaires (n = 1333, minimum age: 20 years, maximum age: 88 years). Quality analysis of the collected data was conducted in order to eliminate from the dataset non-realistic data, after which 1305 (620 women and 685 men) questionnaires (98%) were finally included in the statistical analysis. 

Table 1 reports data of individuals subdivided into the three age groups and the statistical significances between groups. Considering the overall population, the youngest employees were characterized, as expected, by the best anthropometric profile (*p* < 0.001), best sleep hygiene (number of slept hours, *p* = 0.05); nevertheless, they presented the worst lifestyle determinants profile: reduced quality of nutrition (*p* = 0.010, AHA Diet Score), increased perception of stress, fatigue, and stress-related somatic symptoms (*p* < 0.001), a higher percentage of smokers (*p* = 0.022), and the worst perception of job performance (*p* = 0.01). The lifestyle index was the worst (*p* < 0.001), particularly in its stress component (*p* < 0.001). Contrasts, considering the three age groups, are indicated in the table. 

The differences observed considering the three age groups (overall population) were evident also considering only women (Table 2) or only men (Table 3) subjects subdivided into the three age subgroups. In women, the youngest employees were further characterized by a slightly (*p* = 0.05) reduced perception of health quality and a clear (*p* < 0.001) reduced perception of job performance. In men, the youngest employees were further characterized, as expected, by a greater volume of vigorous physical activity.

**Figure 1:**
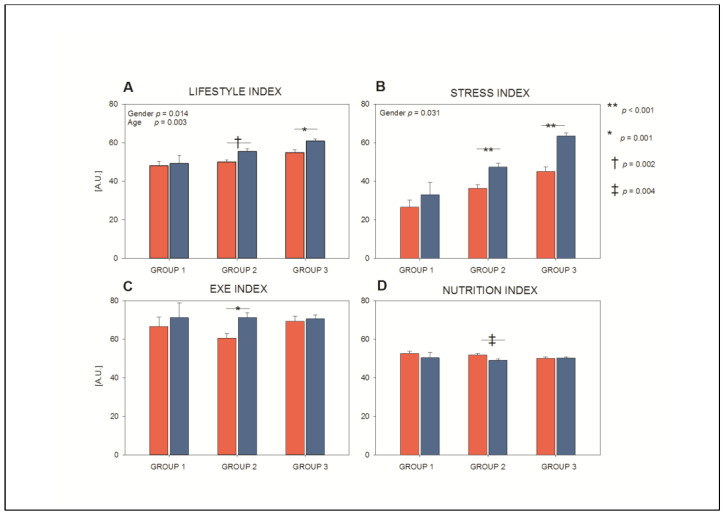
Lifestyle (panel (**A**)), stress (panel (**B**)), exercise (panel (**C**)), and nutrition (panel (**D**)) indexes in the three age groups in women (red bars) and men (blue bars) employees. Note that the lifestyle index (which simultaneously considers the contribution of stress, nutrition, and exercise habits) best depicts the differences due to age and gender and that the youngest (both men and women) employees presented the worst lifestyle index, particularly in its stress component. EXE = exercise.

Figure 1 shows that considering the overall population, the youngest (both men and women) employees presented the worst lifestyle index (age *p* = 0.003), particularly in its stress component. The lifestyle index, which simultaneously considers the contribution of stress, nutrition, and exercise in determining lifestyle, well evidences the difference in the three considered age groups. Additionally, gender (*p* = 0.014) contribution was present, particularly considering groups 2 and 3, and stress index. This latter index (built combining the perception of stress, fatigue and somatic symptoms related to stress) was lower, i.e., less healthy, in women as compared with men (gender *p* = 0.031), particularly in groups 2 and 3.

## 4. Discussion

Our study offers new information on the possibility to start early prevention of CNCD in young adults through the administration of an anonymous, web-based questionnaire aimed at easily detecting age differences in lifestyle in order to tailor interventions to foster health at the worksite and, consequently, to reduce the future risk of CNCD.

In this study, we observed an influence of age on the lifestyle index: the youngest employees (both men and women) presented the worst lifestyles, particularly in their stress components. This observation, obtained simply using an anonymous web-based questionnaire, may potentially be useful to tailor workplace health-promotion programs and to personalize insurance protocols and services offered to employees.

The workplace may play a pivotal role in the prevention of CNCD, particularly through the possibility of offering educational and intervention programs to promote health in the young adult population. This latter specific age group (in the present study represented by employees of group one) is an ideal population [20] to address prevention and modify lifestyle. Young people, as expected, were generally healthier than elders, presenting better lipid profiles, glucose, and arterial blood pressure levels and, obviously, a reduced incidence of CNCD [15]. The assessment of risk factors, such as arterial pressure level, lipid profile, waist circumferences, BMI, smoking, and plasma glucose levels, represents a necessary step in determining the probability to develop chronic diseases such as diabetes and chronic atherosclerotic diseases. Algorithms to calculate cardiovascular risk are based on risk factors [9,34] and they are very useful when the goal is to determine the risk to develop a major cardiovascular disease in the next 10 years. On the contrary, when the goal is to discover, above all in the young population, elements which may drive toward the possibility to develop CNCD in the long term (more than 10 years), a different approach may be more suitable [15,21,22]. To this aim, focusing on lifestyle may be more appropriate. An unhealthy lifestyle, in fact, generally present before the occurrence of conventional risk factors, may influence their appearance and then, in combination with genetic factors [35], affect CNCD. Lifestyle assessment might then be taken into consideration as a mandatory tool to tailor prevention programs.

We used a simple questionnaire [30] to build a unitary lifestyle index (which ranged from 0 to 100 (higher scores indicating a healthier condition), combining self-reported metrics for diet, exercise, and stress) that may provide information on individual global lifestyles. Such an index may be useful in workplace programs, balancing the need to save costs, to reach a large sample of the population, and to provide personalized feedback. The questionnaire, in fact, although anonymous, provided every single participant with a personalized immediate report based on the provided information. This report, by identifying possibly unhealthy lifestyle components (for instance poor physical activity level or poor quality of nutrition), might motivate the participant to improve this/these lifestyle components even before the appearance of conventional risk factors (for instance, high lipid or arterial pressure levels).

In this study, we observed that the youngest employees were characterized by the worst lifestyle index, particularly in its stress component. Moreover, we observed that the gender differences in Exercise Index and Nutrition Index were most evident in employees aged from 31 to 50 years (suggesting again the importance of age in determining specific differences in lifestyle) and the worst perception of job performance was found in young employees, mainly in women.

Age differences in stress perception and in emotional responses to daily stress are reported in the literature [36]. For instance, during the recent COVID-19 outbreak [37] young adults appeared to have a more pessimistic outlook and reported higher perceived stress levels [37]. A great deal of discussion is present in the psychological literature regarding the link among age, stress, coping strategies, control, responsibility feeling for the management and solution of problems, escapism, and depression. Many factors need to be considered in this regard, and gender may also play a role. The results of our study may not help to disentangle this issue, but may give a contribution to an important practical aspect in workplace health promotion programs: the need to assess stress perception in a simple, scientifically-based, and cost-convenient manner. The feasibility to assess stress perception, simply using a few questions inquiring about stress both from a somatic (asking questions regarding the perception of fatigue and other somatic symptoms such as palpitations or muscular tension) and a cognitive (directly asking about stress perception: “do you feel stressed” [38]) point of view, may provide a simple metric in workplace health promotion interventions [24,25,26].

Of interest is also the finding that the gender differences in Exercise Index and Nutrition Index were most evident in employees aged from 31 to 50 years (group two). This result may be due to the fact that group two included the greatest number of respondents (n = 621), well balanced between women (n = 327) and men (n = 294). Nevertheless, also group three included a consistent number of respondents (n = 594) with a slight prevalence of men (n = 368), but no gender differences in exercise and nutrition habits were evident.

The possibility to have a single unitary Lifestyle Index (ranging from 0 to 100 (higher scores being healthier), which combines self-reported metrics for diet, exercise, and stress grants information on individual global lifestyles. The use of this index allowed us in the present study to unveil important practical information showing, for instance, its improvement with aging. The questionnaire was hosted, as a part of an ongoing initiative to promote a healthy lifestyle, on the website of a non-profit association and participation in this survey was on a voluntary basis. This aspect may suggest that only employees who paid attention to their lifestyle or were actually “curious” about the personalized immediate report filled out this questionnaire, which might imply a selection bias in the cohort included. On the other hand, this observation might suggest that an individual’s interest towards their own health increases with aging in a working population. This information may be important to drive workplace policies and may indicate that workplace health promotion programs and insurance services need to focus their attention to improve the interest towards health and lifestyle in the youngest workers. Insurance services aiming only to detect the presence of diseases or risk factors (typically the classic health check-ups) may be of no interest to young employees who consider themselves as healthy people. Vice versa, a different approach [27,39] focusing on improving lifestyle might be more appropriate and might raise more interest in this subgroup.

To corroborate this issue, we may consider that young employees (group one) presented the highest percentage of smokers and the worst, particularly in men, AHA diet score (which gives information on nutrition “quality”), while, as expected, had the lowest BMI and waist circumference, within normal ranges.

We have to acknowledge some limitations of our study. First, data were obtained by self-reported questionnaires and therefore might be of suboptimal quality. However, the elevated number of respondents and the detailed analysis of data quality we performed may have limited the impact of this possible limitation. In addition, although the questionnaire was completely anonymous, it nevertheless provided each individual participant with personalized immediate feedback based on the filled information. In a previous study we have indeed shown that this approach increased participants’ compliance in imputing reliable data [13,26] in order to obtain a report actually reflecting their condition. Finally, we do not have blood chemistry or hemodynamic parameters in order to define the actual level of cardiovascular risk in the individuals included in our study. The employed questionnaire was designed simply to collect information on lifestyle and on clinical characteristics (such as weight, height, and waist circumference) which might be easily obtained without requiring invasive or costly examinations (blood drawing or physician’s consultation). Although we acknowledge this possible limitation, we believe that our data are reliable enough because, in a previous paper from our group [30], we were able to show that the Lifestyle Index did correlate significantly with key biochemical, hematological, and hemodynamic variables predicting levels of cardiometabolic risk.

## 5. Conclusions

In conclusion, this study shows an important influence of age on lifestyle and stress perception in a working population. Such an influence was disclosed by employing an anonymous, simple web-based questionnaire providing data on lifestyle components (such as nutrition, exercise, smoking, stress, and sleep). This study might contribute to tailoring worksite health promotion intervention and insurance services offered to work people, initiatives which may play an important role to foster health, well-being, and to prevent CNCD starting from a young age.

The practical message of our study is that in young, healthy individuals, to drive the attention to current behavior, to the possibility to foster well-being and improve performance, might foster proactive actions which may grant an “immediate” benefit further to the possibility to reduce CNCD. This “immediate” advantage may represent a strong motivation to adhere to healthy lifestyles and may be of particular interest in individuals with elevated perception of stress and/or of somatic symptoms related to stress (such as the youngest employees in our study) in consideration of the proved role of health habits, in particular exercise, in managing stress.

On the contrary, to focus only on medical parameters (frequently within normal ranges), albeit important, may be insufficient to catch their attention, resulting in maintaining an unhealthy behavior.

## Figures and Tables

**Table 1:** Anthropometric and lifestyle data collected in the three age groups.

Variables	≤30 Years Group 1	31–50 Years Group 2	>50 Years Group 3	Significance *p*
N	90	621	594	
Age (years)	27.30 ± 2.23	42.83 ± 5.64 *	58.08 ± 6.94 *†	<0.001
Weight (Kg)	61.82 ± 11.14	70.68 ± 15.88 *	74.10 ± 13.50 *†	<0.001
BMI (Kg/m^2^)	21.80 ± 2.79	23.84 ± 4.19 *	24.76 ± 3.48 *†	<0.001
Height (cm)	167.96 ± 8.97	171.59 ± 9.30 *	172.57 ± 8.71 *	<0.001
Waist circumference (cm)	77.18 ± 10.85	84.24 ± 14.08 *	91.30 ± 12.32 *†	<0.001
Activity volume (moderate brisk walking) (MET·min/week)	330.77 ± 369.62	366.96 ± 476.71	434.61 ± 475.92 *†	ns
Activity volume (other moderate activities) (MET·min/week)	264.27 ± 299.48	250.71 ± 374.31	281.25 ± 430.63	ns
Activity volume (vigorous) (MET·min/week)	456.89 ± 901.55	424.41 ± 814.57	369.94 ± 742.86	ns
Total Activity volume (MET·min/week)	1051.92 ± 1215.13	1042.08 ± 1308.11	1085.80 ± 1207.46	ns
AHA Diet Score (au)	2.04 ± 1.04	2.17 ± 1.03	2.31 ± 1.02 *†	0.010
Smoke (n (%))	19 (21.1)	96 (15.5)	69 (11.6)	0.022
Short 4SQ score (au)	9.36 ± 7.41	8.01 ± 7.73	5.49 ± 6.51 *†	<0.001
STRESS perception (au)	6.11 ± 2.31	4.96 ± 2.99 *	3.65 ± 2.88 *†	<0.001
FATIGUE perception (au)	5.63 ± 2.43	4.67 ± 2.92 *	3.41 ± 2.86 *†	<0.001
SLEEP (hours per night)	7.09 ± 1.05	6.76 ± 1.12 *	6.69 ± 1.04 *	0.05
Perception of sleep quality (au)	6.73 ± 1.72	6.26 ± 2.11 *	6.19 ± 2.20 *	ns
Perception of HEALTH quality (au)	7.20 ± 1.26	6.87 ± 1.68	6.96 ± 1.48	ns
Perception of JOB PERFORMANCE (au)	3.96 ± 0.66	4.27 ± 0.75 *	4.28 ± 0.80 *	0.01
NUTRITION index (au)	52.01 ± 10.10	50.66 ± 11.47	50.12 ± 11.75	ns
EXERCISE index (au)	67.79 ± 39.85	65.60 ± 42.20	70.03 ± 40.33	ns
STRESS index (au)	28.24 ± 30.32	41.60 ± 34.14 *	56.52 ± 33.81 *†	<0.001
LIFESTYLE INDEX (au)	48.35 ± 17.65	52.40 ± 20.72	58.51 ± 20.44 *†	<0.001

Data are presented as mean ± SD; significance contrast: * vs. group 1; † vs. group 2. Abbreviations: *p* = significance; BMI = body mass index; AHA = American Heart Association; MET = Metabolic Equivalent; 4SQ = Subjective Somatic Stress Symptoms Questionnaire; au = arbitrary units.

**Table 2:** Anthropometric and lifestyle data were collected in women by separately considering the three age subgroups.

Variables	≤30 Years Group 1	31–50 Years Group 2	>50 Years Group 3	Significance *p*
N	67	327	226	
Age (years)	27.22 ± 2.28	42.35 ± 5.68 *	55.84 ± 4.03 *†	<0.001
Weight (Kg)	57.21 ± 7.86	61.25 ± 12.34 *	64.07 ± 10.66 *†	<0.001
BMI (Kg/m^2^)	21.18 ± 2.61	22.37 ± 4.27 *	23.61 ± 3.77 *†	<0.001
Height (cm)	164.33 ± 6.42	165.43 ± 6.54	164.73 ± 5.82	ns
Waist circumference (cm)	74.38 ± 9.77	77.73 ± 12.38 *	85.10 ± 11.92 *†	<0.001
Activity volume (moderate brisk walking) (MET·min/week)	300.49 ± 334.77	369.72 ± 504.54	431.20 ± 450.16	ns
Activity volume (other moderate activities) (MET·min/week)	241.73 ± 289.38	255.99 ± 400.30	286.30 ± 432.53	ns
Activity volume (vigorous) (MET·min/week)	312.24 ± 576.89	303.36 ± 723.93	278.05 ± 680.20	ns
Total Activity volume (MET·min/week)	854.46 ± 885.52	929.07 ± 1302.77	995.55 ± 1220.01	ns
AHA Diet Score (au)	2.12 ± 0.99	2.31 ± 1.06	2.44 ± 1.08	ns
Smoke (n (%))	13 (19.4)	40 (12.2)	35 (15.5)	ns
Short 4SQ score (au)	10.30 ± 7.24	9.14 ± 7.85	7.25 ± 7.46 *†	0.003
STRESS perception (au)	6.22 ± 2.30	5.28 ± 2.99 *	4.63 ± 3.07 *†	<0.001
FATIGUE perception (au)	5.75 ± 2.48	5.11 ± 2.97	4.44 ± 3.09 *†	0.002
SLEEP (hours per night)	7.18 ± 1.01	6.85 ± 1.15 *	6.66 ± 1.09 *†	0.003
Perception of sleep quality (au)	6.70 ± 1.86	6.18 ± 2.15	6.15 ± 2.29	ns
Perception of HEALTH quality (au)	7.22 ± 1.33	6.76 ± 1.75 *	7.00 ± 1.50	0.05
Perception of JOB PERFORMANCE (au)	3.90 ± 0.67	4.28 ± 0.76 *	4.34 ± 0.68 *	<0.001
NUTRITION index (au)	52.55 ± 9.30	51.88 ± 12.01	49.95 ± 12.98	ns
EXERCISE index (au)	66.64 ± 39.58	60.49 ± 43.36	69.24 ± 40.56†	ns
STRESS index (au)	26.59 ± 30.18	36.40 ± 33.24 *	45.19 ± 34.61 *†	<0.001
LIFESTYLE INDEX (au)	48.05 ± 17.73	50.00 ± 20.41	54.98 ± 20.46 *†	0.008

Data are presented as mean ± SD; significance contrast: * vs. group 1; † vs. group 2. Abbreviations: *p* = significance; BMI = body mass index; AHA = American Heart Association; MET = Metabolic Equivalent; 4SQ = Subjective Somatic Stress Symptoms Questionnaire; au = arbitrary units.

**Table 3:** Anthropometric and lifestyle data were collected in male individuals, by separately considering the three age subgroups.

Variables	≤30 Years Group 1	31–50 Years Group 2	>50 Years Group 3	Significance *p*
N	23	294	368	
Age (years)	27.52 ± 2.08	43.35 ± 5.56 *	59.47 ± 7.93 *†	<0.001
Weight (Kg)	75.26 ± 7.94	81.18 ± 12.42 *	80.26 ± 11.17 *	0.05
BMI (Kg/m^2^)	23.64 ± 2.49	25.47 ± 3.44 *	25.47 ± 3.07 *	0.028
Height (cm)	178.52 ± 6.74	178.44 ± 6.73	177.39 ± 6.39	ns
Waist circumference (cm)	85.74 ± 9.59	92.83 ± 11.34 *	95.51 ± 10.72 *†	<0.001
Activity volume (moderate brisk walking) (MET·min/week)	418.95 ± 453.37	363.90 ± 444.55	436.71 ± 491.65	ns
Activity volume (other moderate activities) (MET·min/week)	329.91 ± 324.91	244.83 ± 343.70	278.15 ± 430.01	ns
Activity volume (vigorous) (MET·min/week)	878.26 ± 1482.41	559.05 ± 886.79	426.37 ± 774.36 *†	0.013
Total Activity volume (MET·min/week)	1627.13 ± 1776.56	1167.78 ± 1304.77	1141.23 ± 1197.99	ns
AHA Diet Score (au)	1.83 ± 1.23	2.00 ± 0.97	2.23 ± 0.98 *†	0.004
Smoke (n (%))	6 (26.1)	56 (19.0)	34 (9.2)	0.001
Short 4SQ score (au)	6.61 ± 7.38	6.75 ± 7.42	4.42 ± 5.59 †	<0.001
STRESS perception (au)	5.78 ± 2.35	4.60 ± 2.94 *	3.05 ± 2.59 *†	<0.001
FATIGUE perception (au)	5.30 ± 2.30	4.18 ± 2.78 *	2.78 ± 2.52 *†	<0.001
SLEEP (hours per night)	6.83 ± 1.15	6.65 ± 1.09	6.70 ± 1.02	ns
Perception of sleep quality (au)	6.83 ± 1.23	6.35 ± 2.06	6.22 ± 2.15	ns
Perception of HEALTH quality (au)	7.13 ± 1.05	7.00 ± 1.59	6.94 ± 1.48	ns
Perception of JOB PERFORMANCE (au)	4.13 ± 0.62	4.26 ± 0.74	4.25 ± 0.87	ns
NUTRITION index (au)	50.37 ± 12.36	49.04 ± 10.52	50.23 ± 10.86	ns
EXERCISE index (au)	71.12 ± 41.36	71.28 ± 40.18	70.52 ± 40.23	ns
STRESS index (au)	33.05 ± 30.89	47.39 ± 34.26 *	63.48 ± 31.38 *†	<0.001
LIFESTYLE INDEX (au)	49.28 ± 17.87	55.56 ± 20.73	60.92 ± 20.10 *†	0.001

Data are presented as mean ± SD; significance contrast: * vs. group 1; † vs. group 2. Abbreviations: *p* = significance; BMI = body mass index; AHA = American Heart Association; MET = Metabolic Equivalent; 4SQ = Subjective Somatic Stress Symptoms Questionnaire; au = arbitrary unit.

## Data Availability

Data will be available on justified request. We have not uploaded the data because they are part of an ongoing study on lifestyle modification program in the workplace and other papers may be prepared using them.
